# Correction: Selected Articles in Blood Research, volume 60

**DOI:** 10.1007/s44313-025-00083-5

**Published:** 2025-05-26

**Authors:** Blood Research

**Affiliations:** Seoul, Republic of Korea

**Correction** **:** **Blood Res 60, 8 (2025); 60, 10 (2025); 60, 12 (2025)**

**https://doi.org/10.1007/s44313-024-00053-3**

**https://doi.org/10.1007/s44313-025-00059-5**

**https://doi.org/10.1007/s44313-025-00060-y**

The original publications of the following articles did not contain a structured abstract. The correct abstracts are provided in this erratum, and the original articles have been updated.

*Blood Res.* 60, 8 (2025). 10.1007/s44313-024-00053-3

**Abstract**

**Purpose:**

To report the clinical course and nailfold capillaroscopic changes in a patient with polycythemia vera (PV) and acrocyanosis, underscoring the potential of ruxolitinib to modulate microvascular dysfunction associated with myeloproliferative neoplasms.

**Methods:**

We present the case of a 78-year-old woman with PV who developed pronounced acrocyanosis and abnormal nailfold capillaroscopic findings during treatment with hydroxyurea. Due to suboptimal symptom control and hematological side effects, the therapy was switched to ruxolitinib. Clinical assessments and nailfold capillaroscopy were conducted before and after the therapeutic change.

**Results:**

Following the initiation of ruxolitinib, the patient experienced marked clinical improvement, including resolution of acrocyanosis and normalization of hematologic parameters. Follow-up capillaroscopy revealed a significant reduction in microvascular abnormalities, with restored architectural organization and only mild residual capillary tortuosity.

**Conclusion:**

Ruxolitinib may offer dual therapeutic advantages in PV by effectively managing the hematologic burden of disease and improving microvascular symptoms such as acrocyanosis. Nailfold capillaroscopy may serve as a valuable non-invasive tool for assessing and monitoring microcirculatory involvement in patients with myeloproliferative neoplasms.

*Blood Res.* 60, 10 (2025). 10.1007/s44313-025-00059-5

**Abstract**

**Purpose:** The efficacy and safety of polatuzumab vedotin combined with rituximab, cyclophosphamide, doxorubicin, and prednisolone (pola-R-CHP) in patients aged ≥ 80 years with untreated diffuse large B-cell lymphoma (DLBCL) remain largely unexplored.

**Methods:** In this study, we administered a reduced-dose pola-R-CHP regimen to 38 patients with DLBCL aged > 80 years. Extending the findings of the POLARIX trial in this older individuals’ cohort, we conducted a retrospective analysis to assess the efficacy and safety of the treatment in a real-world clinical setting.

**Results:** After 12 months, the overall and progression-free survival rates were 86.2% (95% confidence interval [CI]: 70.0–94.0) and 78.5% (95% CI: 59.2–89.5), respectively. Although the incidence of febrile neutropenia was relatively high (32%), an increased risk was observed in patients with an average relative dose intensity of < 70%, even with reduced treatment intensity. Notably, none of the patients required a dose reduction of polatuzumab vedotin owing to peripheral neuropathy.

**Conclusion **Therefore, our findings indicate that a reduced-dose pola-R-CHP regimen may be a viable and effective treatment option for older patients newly diagnosed with DLBCL.

*Blood Res.* 60, 12 (2025). 10.1007/s44313-025-00060-y

**Abstract**

**Purpose**

Allogeneic stem cell transplantation (allo-SCT) is a potentially curative treatment option for patients with relapsed or refractory lymphoid malignancies. However, the prognostic factors influencing survival outcomes in these patients remain poorly defined. This study aimed to evaluate the clinical variables associated with progression-free survival (PFS) and overall survival (OS) in patients undergoing allo-SCT for lymphoid malignancies.

**Methods**

We analyzed 58 patients who underwent allo-SCT for lymphoid malignancies, including B-cell lymphoma (BCL, *n* = 20), Hodgkin lymphoma (*n* = 3), multiple myeloma (*n* = 9), natural killer/T-cell lymphoma (NK/TCL, *n* = 4), and T-cell lymphoma (TCL, *n* = 22). Clinical factors such as HLA matching and post-transplant response status were assessed for their association with survival outcomes using univariate and multivariate analyses.

**Results**

The median PFS and OS were 27.4 months and 30.6 months, respectively. In univariate analysis, both HLA matching and complete remission (CR) status after transplantation were associated with superior PFS and OS. However, multivariate analysis identified only post-transplant CR as an independent predictor of improved survival. Subgroup analysis revealed that HLA matching was significantly associated with better PFS in patients with BCL and NK/TCL, and with better OS only in BCL. Post-transplant CR was consistently associated with improved PFS and OS across BCL, NK/TCL, and TCL subtypes.

**Conclusion**

Post-transplant response is a key prognostic factor influencing survival in allo-SCT for lymphoid malignancies. Achieving complete remission after transplantation may help guide post-SCT management and risk-adapted therapeutic strategies.

